# Estimating genotypic richness and proportion of identical multi-locus genotypes in aquatic microalgal populations

**DOI:** 10.1093/plankt/fbac034

**Published:** 2022-07-16

**Authors:** Ingrid Sassenhagen, Deana L Erdner, Bryan C Lougheed, Mindy L Richlen, Conny SjÖqvist

**Affiliations:** Department of Ecology and Genetics/Limnology, Uppsala University, Uppsala, Sweden; Marine Science Institute, The University of Texas at Austin, Port Aransas, TX, USA; Department of Earth Sciences, Uppsala University, Uppsala, Sweden; Biology Department, Woods Hole Oceanographic Institution, Woods Hole, MA, USA; Environmental and Marine Biology, Åbo Akademi University, Turku, Finland

**Keywords:** genotypic richness, proportion of identical multi-locus genotypes, microalgal blooms, modeling, python

## Abstract

The majority of microalgal species reproduce asexually, yet population genetic studies rarely find identical multi-locus genotypes (MLG) in microalgal blooms. Instead, population genetic studies identify large genotypic diversity in most microalgal species. This paradox of frequent asexual reproduction but low number of identical genotypes hampers interpretations of microalgal genotypic diversity. We present a computer model for estimating, for the first time, the number of distinct MLGs by simulating microalgal population composition after defined exponential growth periods. The simulations highlighted the effects of initial genotypic diversity, sample size and intraspecific differences in growth rates on the probability of isolating identical genotypes. We estimated the genotypic richness for five natural microalgal species with available high-resolution population genetic data and monitoring-based growth rates, indicating 500 000 to 2 000 000 distinct genotypes for species with few observed clonal replicates (<5%). Furthermore, our simulations indicated high variability in genotypic richness over time and among microalgal species. Genotypic richness was also strongly impacted by intraspecific variability in growth rates. The probability of finding identical MLGs and sampling a representative fraction of genotypes decreased noticeably with smaller sample sizes, challenging the detection of differences in genotypic diversity with typical isolate numbers in the field.

## INTRODUCTION

Free-living microalgal populations characteristically comprise huge numbers of cells (up to 1×10^6^ cells/L e.g. Heil and Steidinger, 2009; Leblanc *et al.*, 2012; Lebret *et al.*, 2012; Ryan *et al.*, 2017). Although the majority of microalgal species reproduce asexually over most of the growth season (Figueroa *et al.*, 2018; Steidinger and Garces, 2007), only a few population genetic studies have found identical multi-locus genotypes (MLGs) among cultivated strains (Ruggiero *et al.* 2017). Instead, most studies have identified high genotypic richness in microalgal species, reflecting ecological and evolutionary selection pressures (Rengefors *et al.*, 2017). Understanding the level of genotypic richness is particularly important given the influence and degree of anthropogenic global change (Litchman *et al.*, 2012; Reusch and Boyd, 2013), as it may act as a proxy for adaptive evolution (Schlüter *et al.*, 2016).

The paradox of frequent asexual reproduction but a low number of observed identical MLGs has raised questions about the actual level of genotypic richness in natural microalgal populations. Although certain comparisons between bacterioplankton and eukaryotic microalgae are not appropriate due to their different life cycle strategies, extensive intraspecific diversity has been demonstrated in marine cyanobacteria (Johnson *et al.*, 2006; Paul *et al.*, 2010) as a result of niche partitioning and ecotype-specific genomes. Given that marine microorganisms, pro- and eukaryotic, inhabit the same environment and face similar selection pressures, do we actually sample a significant proportion of the genotypic diversity in microalgal populations, or are our sample sizes in cultivation-based studies simply too small for observing identical MLGs? Answering this question is highly relevant for understanding and interpreting any findings about microalgal diversity.

Given the high number of microalgal cells and the dependence on culturing microalgal strains for genetic fingerprinting, analyzing a significant proportion of cells from any microalgal population has remained challenging despite major advances in single-cell and high-throughput sequencing methods. To circumvent these problems, previous studies have tried to mathematically estimate genotypic richness in microalgal populations (Rynearson and Armbrust, 2005; Watts *et al.*, 2013). The previously utilized Chao1 index (Chao, 1987) relies on a capture-recapture approach, which is widely used by field ecologists to estimate species richness. This index attempts to incorporate heterogeneity of capture probability, but the potentially large differences in abundance among distinct clonal lineages can lead to an underestimation of the actual genotypic richness. This issue would be amplified, if we do not sample a significant proportion of all present genotypes.

To overcome some of these limitations, we developed a simple demographic model in order to explore the impact of intraspecific variability in growth rates, resulting in varying genotype specific abundances, and sample size on the probability of isolating identical MLGs from a pre-defined microalgal population. By solely focusing on the relatively short exponential growth period leading to the formation of microalgal blooms, we were able to neglect factors such as sexual recombination and dispersal. Based on these simulations, genotypic richness in specific populations of different microalgae was estimated by incorporating species-specific monitoring data and observed proportions of identical MLGs from published population genetic studies. Furthermore, the effect of genetic marker choice on these simulations and comparisons with Chao1 diversity estimates are discussed.

**Glossary Box:** Important terminology utilized in the model and throughout the text.

Term	Definition
Multi-locus genotype (MLG)	A microalgal strain that is characterized by a certain set of genetic loci distinguishing it from other strains.
Generic model species	A fictive microalgal species with idealized characteristics created to illustrate the impact of different parameters in the model.
Natural species	An actual microalgal species that has been described and investigated in several studies providing in-situ cell counts and population genetic data.
Population	Microalgal cells of the same species that inhabit together the same geographic space and are genetically related to each other.
Mean growth rate (model parameter μ)	Calculated from several population growth rates established in different habitats or at different time points.
Intraspecific variability in growth rates (model parameter σ)	The standard deviation of the mean growth rate (μ) of the microalgal population. This variability in growth rates results from different adaptations, physiological characteristics and micro-environments of the individual MLGs.
Sample size (model parameter)	The number of microalgal cells that were isolated and fingerprinted in a study.
Days (model parameter)	Number of days with exponential growth resulting in formation of a microalgal blooms.
Cell (model parameter)	The initial number of cells per MLG at the beginning of the exponential growth period.

## MATERIAL AND METHODS

A simplistic demographic model was constructed in Python 3.7.6 using NumPy (Harris *et al.*, 2020) to assess the probability of isolating identical MLGs in field studies by simulating microalgal population composition after defined exponential growth periods. The impact of the initial number of distinct genotypes, the intraspecific variability in growth rates and the sample size on these simulations was investigated using generic model species (see Glossary Box for definitions). Afterwards, the model was utilized to estimate genotypic richness in five natural microalgal species based on growth rates from monitoring cell counts and published population genetic data. The estimates from the simulations were compared to Chao1 indices of genotypic richness. Finally, the effect of the amount of genetic loci on the observed number of distinct genotypes was investigated using a rarefaction analysis to illuminate our definition of MLGs.

### General set-up of the simulation

**Fig. 1:**
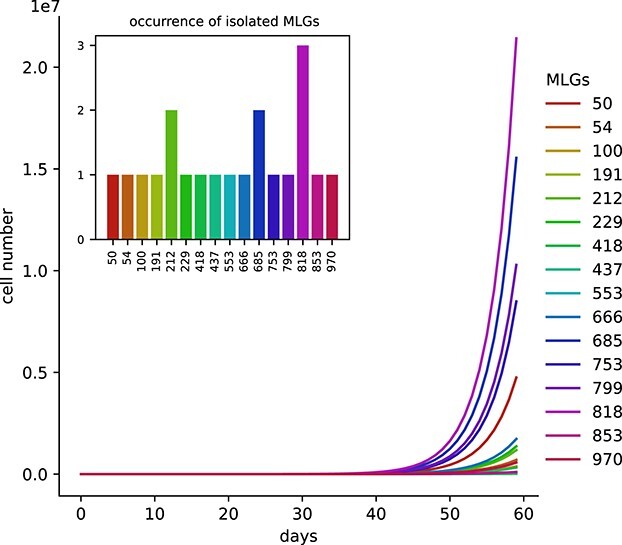
Visualization of the increase in cell numbers of multi-locus genotypes (MLG) over 60 days and the occurrence of identical MLGs among the isolates. For this model run, 1 000 distinct genotypes, a sample size of 20 isolates, and a growth rate distribution with μ = 0.2 and σ = 0.04 were chosen. Every MLG was assigned a number from 0 to 999.

In the presented simulation, the number of identical MLGs was defined as the differences between the sample size (number of isolates) and the observed number of distinct MLGs. Individual growth rates were randomly sampled with weighting from a constant growth rate function characterized by a normal distribution with mean (μ) and an estimated standard deviation representing variation in intraspecific growth rates (σ), and assigned to individual MLGs of a pre-defined population. The proportion of each genotype in the population was calculated after a defined number of days (t) with exponential growth (Fig. 1) following the formula N_t_ = N_0_ * e^μ*t^. This approach does not consider mutations or sexual recombination, as most microalgal species only sexually reproduce at the end of the growth season or even every few years, but not during the exponential growth phase that leads to the formation of blooms (Figueroa *et al.*, 2018). Furthermore, the simulations attempt to shed light on observations from population genetic studies largely based on microsatellite data, which are unlikely to mutate within the investigated time frame. Isolates were picked based on weighted proportions of genotypes and sample size (Fig. 1) using the random.choices method in python. To increase statistical power, the picking procedure was repeated 100 times. Furthermore, the entire procedure starting from the assignment of individual growth rates to each MLG was repeated 10 times. The probability of observing the same genotype more than once was calculated for each of the simulations by taking the mean of the 1000 (100 picking events × 10 repeats) simulation outputs.

As this modeling environment allows constructing an idealized experimental design, we were able to separately quantify the impact of varying individual variables upon the probability of isolating identical MLGs by keeping other variables constant. The set-up of the model allows testing ranges in initial genotypic richness (number of distinct MLGs), intraspecific differences in growth rates and sample size. In each model run, the combined effect of variation in two of the aforementioned variables on the probability of isolating identical MLGs was tested, while the third variable was held constant. Starting cell concentrations of each MLG can be set to 1 or specified as a range (e.g. 1 to 100), from which the initial cell number of each genotype is randomly selected, depending on the ecology and life cycle of the simulated microalgal species. It might e.g. be appropriate to assume one cell per MLG for microalgal populations germinating from sexually produced resting stages, while populations without cyst formation might comprise several cells per MLG at the beginning of the growth season.

### Simulations using generic model species

To investigate the general effects of varying initial genotypic diversity, intraspecific variability in growth rates and sample sizes on the probability of isolating identical MLGs, two generic model species were utilized. A slow-growing model species was characterized by μ = 0.15 and an exponential growth period of 60 days, while for the second, fast-growing model species μ = 0.4 and an exponential growth period of 20 days were assumed. The initial cell number per genotype was set to one and the standard deviation in growth rates was assumed to be σ = 0.02. We ran 88 unique simulations based on all possible combinations involving eleven discrete initial population sizes ranging from 4000 to 100 000 unique genotypes, and eight discrete sample sizes ranging from 20 to 500 isolates. To test the effect of intraspecific differences in growth rates, the simulations were repeated for the same model species implementing σ = 0.08, which represented strongly increased differences in growth rates among genotypes for both generic model species. The simulations were also run with initial cell numbers ranging from one to 10 000 cells per genotype to investigate the effect of uneven initial cell concentrations.

### Simulations using published data from natural microalgal species

The probability of isolating identical MLGs was also simulated for five natural microalgal species utilizing monitoring, growth rate and population genetic data from previously published studies. This model approach is particularly dependent on population genetic studies with very high sample sizes (>100 isolates) of the same genetic population, which identified at least one MLG twice using a set of genetic markers. The required information was available for the dinoflagellate *Alexandrium catenella*^1^ in the submerged, tidal kettle pond called “Salt Pond” in the Nauset Marsh System on Cape Cod, Massachusetts (USA) (Richlen *et al.*, 2012)*, Alexandrium ostenfeldii* in the Baltic Sea (Jerney, 2020), the diatoms *Ditylum brightwellii* in Puget Sound, Washington (USA) (Rynearson and Armbrust, 2005)*, Skeletonema marinoi* in Gullmar Fjord on the Swedish west coast in 2008 (Godhe and Härnström, 2010) and *Pseudo-nitzschia multistriata* from the Gulf of Naples (Italy) (Ruggiero *et al.*, 2017; Tesson *et al.*, 2014).

**Fig. 2:**
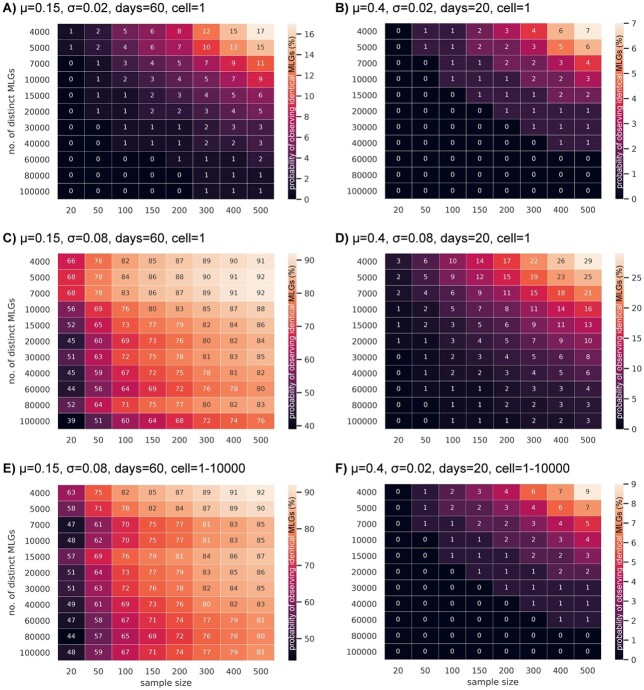
Heatmaps illustrating the probability (%) of observing identical MLGs, indicated by the colour gradient, depending on the sample size (i.e. number of fingerprinted isolates) and the initial number of distinct MLGs at the beginning of the bloom. Please, observe the different scale bars in each sub-figure. Simulations were run for generic model species characterized by different mean growth rates (μ), different intraspecific variability in growth rates (σ), different number of days with exponential growth (days), and different amount of initial cell concentrations per genotype (cell). A) Slow-growing and B) fast-growing species with low intraspecific variability and initially one cell per genotype. C) Slow-growing and D) fast-growing species with high intraspecific variability and initially one cell per genotype. E) Slow-growing species with high intraspecific variability and heterogeneous initial cell numbers per genotype. F) Fast-growing species with low variability and heterogeneous initial cell numbers per genotype.

The observed proportion of identical MLGs in these population genetic studies differed noticeably among the selected species. The study by Ruggiero *et al.* (2017) of the diatom *P. multistriata* observed by far the highest proportion of identical MLGs (81% of 279 isolates), while only 0.65% of 154 isolates from *S. marinoi* were identical in 2008 (Godhe and Härnström, 2010). The cumulative number of MLGs continuously increased throughout the sampling period in *A. catenella* (Richlen *et al.*, 2012), *P. multistriata* (Tesson *et al.*, 2014) and *D. brightwelli* (Rynearson and Armbrust, 2005) resulting in 149, 393 and 496 distinct MLGs in respective studies (Fig. 2; Rynearson and Armbrust, 2005).

For the dinoflagellate *A. catenella*, cell concentrations and length of the exponential growth period in Salt Pond were collected from Crespo *et al.* (2011), Ralston *et al.* (2014), Petitpas *et al.* (2015), Brosnahan *et al.* (2017) and Choi *et al.* (2017). Information about *D. brightwellii* was gathered from Koester *et al.* (2010) and the HELCOM ICES database. Growth rates for *S. marinoi* were calculated from abundance data collected from 2010 to 2019 at the monitoring sites BY31 LandsortsDJ and REFM1V1 in the Baltic Sea (SMHI), and counts in Godhe *et al.* (2016). The required data for *P. multistriata* were provided by Zingone and Montresor (pers. communication). Data about *A. ostenfeldii* were gathered from Kremp, Rengefors, and Montresor (2009) and Jerney *et al.* (2019).

Population growth rates were calculated based on these monitoring field data using the equation μ = (ln N_t_—ln N_0_)/t. Mean (μ) and standard deviation (σ) of growth rates for each species were calculated using R base from data collected at different locations and/or time points. Normal distribution of different growth rates measured in the same species was tested with the Shapiro–Wilk test in the R package ggpubr. The mean population growth rates of the selected natural microalgal species ranged from 0.117 to 0.517 (Table I). The dinoflagellate *A. catenella* required on average 60 days to reach maximum cell concentrations (Brosnahan *et al.*, 2017; Crespo *et al.*, 2011; Ralston *et al.*, 2014), while the fast-growing diatom species *P. multistriata* formed blooms within 14 days (Zingone and Montresor, pers. communication). The intraspecific variability in growth rates also differed substantially among species. In *A. catenella*, the standard deviation of the gathered growth rates from environmental monitoring data was 0.0277, while the standard deviation in *A. ostenfeldii* was 0.06. The highest intraspecific variability in growth rates (σ = 0.178) was found in *P. multistriata*.

**Table I:** Model parameters of selected microalgal species. μ = mean growth rate, σ = standard deviation, growth period = number of days with exponential growth until maximum cell densities are reached, sample size = number of isolates, number of identical MLGs = sample size—# distinct MLG, number of distinct genotypes based on simulation, standard deviation for the estimation of distinct genotypes, number of microsatellites employed to fingerprint the microalgal species

Species	μ	σ (Growth rate)	Growth period (days)	Growth ref.	Sample size	# Identical MLGs	% Identical MLGs	Popgen ref.	# Simulated genotypes	σ (# sim. genotypes)	Chao1	ACE	# Microsats
*Alexandrium ostenfeldii*	0.15	0.06	40	Kremp *et al.*, 2009, Jerney *et al.*, 2019	178	2	1.1	Jerney 2020	2 167 803	550 866–9 634 413	5 193 ± 2485.93	7 832 ± 1.41	9
*Alexandrium catenella 2006*	0.2	0.0277	60	Crespo *et al.*, 2011, Ralston *et al.*, 2014, Petitpas *et al.*, 2015, Brosnahan *et al.*, 2017, Choi *et al.*, 2017	101	19	18.8	Richlen *et al.*, 2012	1964	1909–2 892	392.63 ± 127.96	455.18 ± 6.14	4
*A. catenella 2007*	0.2	0.0277	60		91	14	15		2 582	1908–4 325	402.43 ± 140.034	455.94 ± 4.86	4
*Ditylum brightwellii*	0.18	0.05	20	Koester *et al.*, 2010, HELCOM ICES	587	106	18	Rynearson and Armbrust, 2005	4 095	3 650–4 592	2 400 ± 340	2 700 ± 250	3
*S. marinoi*	0.117	0.04	40	SMHI, Godhe *et al.*, 2016	154	1	0.7	Godhe & Harnström, 2010	181 332	71 927–730 428	5 891 ± 3017.89	11 781 ± 99.84	8
*P. multistriata 2008*	0.517	0.178	14	Zingone and Montresor, pers. Communication	157	53	34	Tesson *et al.*, 2014	10 057	5 933–16 380	676.14 ± 235.28	568.97 ± 5.17	7
*P. multistriata 2009*	0.517	0.178	14		193	57	29		17 480	8 911–28 959	1073.88 ± 354.92	1103.68 ± 6.62	7
*P. multistriata 2010*	0.517	0.178	14		162	8	4		493 496	224 724–820 441	1918.18 ± 720.99	3597.56 ± 19.45	7
*P. multistriata pre-bloom 2013*	0.517	0.178	14		73	6	8.2	Ruggiero *et al.*, 2017	68 013	27 867–82 257	328.43 ± 114.81	407.58 ± 2.43	22
*P. multistriata summer bloom 2013*	0.517	0.178	14		133	15	11.3		76 142	37 335–137 012	1117.17 ± 419.84	1 430 ± 7.04	22
*P. multistriata autumn bloom 2013*	0.517	0.178	14		279	227	81.4		2 873	2 257–3 839	338 ± 163.09	291.33 ± 8.75	22

### Curve-fitting and extrapolation of the number of genotypes

To estimate the number of distinct genotypes in natural microalgal populations, the probability of observing identical MLGs assuming species-specific growth rates with μ and σ, and a set range of population sizes was extracted from the 1000 isolation events for the sample size reported in the population genetic studies. The mean and the standard deviation (σ = 68.27% of values from an assumed normal distribution) were calculated for each population size across the 1 000 replicates. Power-functions were fitted to the mean modeled probabilities of picking identical MLGs, and the means + and—one standard deviation (σ) using the scipy.optimize.curve_fit function in Python 3.7.6 (Virtanen *et al.*, 2020). Based on these functions, the number of genotypes for each microalgal population was calculated considering reported proportions of identical MLGs in the population genetic studies.

### Genotypic richness estimates with Chao1 and ACE

For comparison with the estimated number of distinct MLGs from the simulations genotypic richness estimates based on Chao1 and ACE indices were calculated for all microalgal species. When the number of identical MLGs in the selected natural microalgal populations was not specified in the publication, unique genotypes were identified using the “distinct” function in the R package dplyr (Wickham *et al.*, 2020). Isolates with null alleles were removed for this analysis. Based on counts of occurrence of each MLG, the number of unseen genotypes in the natural microalgal populations was extrapolated and added to the observed genotype richness using bias-corrected Chao1 and ACE indices with the estimateR function in the R package vegan (Oksanen *et al.*, 2016). These indices (Chao, 1987; Chao and Lee, 1992; O’Hara, 2005) are based on the capture-recapture principle and represent estimates for the lower bound of genotypic richness.

To assess the accuracy of Chao1 indices of genotypic richness in microalgal populations, mean Chao1 indices were also calculated for simulated populations of generic species with known numbers of distinct MLGs and a range of different sample sizes. These calculations were based on the same simulations as described above, involving a pre-defined set of MLGs (4000–100 000 distinct MLGs) with randomly assigned individual growth rates from a constant growth rate function defined by μ and σ, and a defined exponential growth period. For comparability with previous simulations, an intermediate average growth rate (μ) of 0.25 with an exponential growth period of 30 days, standard deviations among individual growth rates (σ) of 0.02 and 0.08, and varying initial cell numbers of 1 to 1000 cells per MLG were chosen. A number of individual cells corresponding to the selected sample size (20–500 isolates) was repeatedly isolated and identical MLGs were identified. Based on the occurrence of distinct MLGs, the number of unseen genotypes was extrapolated with the bias-corrected Chao1 index using the python package skbio.diversity.

### Number of distinct genotypes depending on the number of observed loci

To assess the increase in the number of distinct genotypes depending on the number of chosen loci of a genetic marker, a rarefaction analysis was applied to the yearly microsatellite data sets of the diatom *P. multistriata* from Tesson *et al.* (2014). This dataset was chosen due to the absence of null alleles, allowing straightforward identification of distinct genotypes and identical MLGs, and the intermediate number of investigated loci. The number of distinct genotypes based on only two loci was identified for all possible combinations of two microsatellite loci from the available seven loci using the R packages base and dplyr. The same procedure was repeated for increasingly large sets of microsatellite loci up to all seven that were available. The effect of increasing the number of loci on the mean observed amount of genetically distinct genotypes was visualized using the R package ggplot2 (Wickham, 2016).

## RESULTS

### Simulations for generic model species

**Fig. 3:**
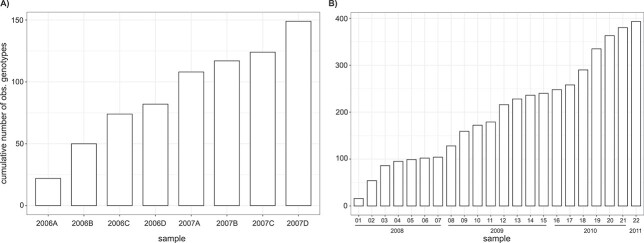
Cumulative number of observed genotypes in *Alexandrium catenella* (A) and *Pseudo-nitzschia multistriata* (B) across samples.

To assess the impact of variable initial genotypic richness, intraspecific differences in growth rates, and sample size, the probability of observing identical MLGs was modeled for generic slow-growing and fast-growing algal species. When the intraspecific variability in growth rates (σ) represented 0.02, and the same sample sizes and number of distinct genotypes were assumed, the probability of observing identical MLGs was very low in both generic species (Fig. 3A and B). In contrast, when assuming a much higher intraspecific variability in growth rates (σ = 0.08), the probability of observing identical MLGs strongly increased in both species. In particular in the slow-growing species, a large proportion of the isolates (>40%) represented identical MLGs in samples of any size and the probability remained very high even with larger numbers of distinct genotypes (Fig. 3C). In the fast-growing species, the probability of observing identical MLGs also increased over all, but remained <10% when the initial number of distinct MLGs exceeded 20 000 (Fig. 3D). Furthermore, this simulation also highlighted the increased unpredictability of final proportions of genotypes in a scenario with very high intraspecific variability in growth rates including extinction of some lineages. These simulations show that the probability of observing identical MLGs is strongly impacted by intraspecific variability in growth rates in this model, especially in slow-growing microalgal species.

**Fig. 4:**
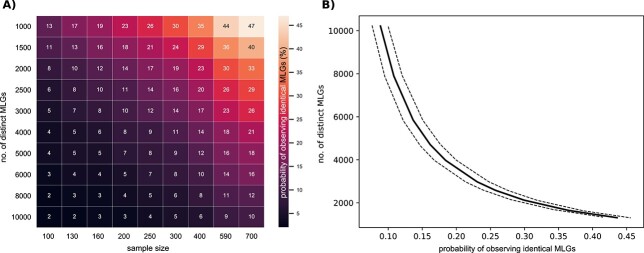
A) Heatmap visualizing the probability (%) of observing identical MLGs of *Ditylum brightwelli*, indicated by the color gradient, depending on the initial number of distinct MLGs and the sample size. μ = 0.18, σ = 0.05, initial cell number per genotype = 1–100, exponential growth period = 20 days. B) Number of *D. brightwelli* genotypes as a function of the modeled probability to pick identical MLGs, assuming a sample size of 590 isolates and an average growth rate of 0.18 with a standard deviation of 0.05. Solid line indicates the mean (R^2^ = 0.99), while dashed lines indicate the standard deviation (σ).

The probability of observing identical MLGs did not change noticeably when varying the number of initial cells per genotype in populations with very high nor with very low intraspecific variability in growth rates (Fig. 3E and F). However, in populations with very high variability in growth rates, a wide range of initial cell numbers (1–10 000) per genotype slightly balanced the occurrence of outlier genotypes with extremely high growth rates and the aforementioned unpredictability of final proportions of genotypes (Fig. 3E).

### Simulations for natural species

The probability of observing identical MLGs was also simulated for five natural microalgal species taking their reported growth rates and exponential growth periods into consideration. As an example, the probability of observing identical MLGs in the different scenarios was visualized for the diatom *D. brightwellii,* as this species displays intermediate growth rates and intraspecific variability. The probability of observing identical MLGs increased with sample size and decreased with the number of distinct genotypes in the population (Fig. 4A). To estimate the genotype richness of *D. brightwellii* in Puget Sound, a power function was fitted to the modeled probabilities of observing identical MLGs depending on the number of genotypes (Fig. 4B), selecting the reported sample size of 590 isolates and an average growth rate of 0.18 with a standard deviation of 0.05. Based on this function and 18% of identical MLGs detected by three microsatellite markers (Rynearson and Armbrust, 2005), we estimated the genotypic richness to be 4095 (3651 < σ < 4592) (Table I).

Based on this approach, the estimated genotypic richness differed substantially among the five selected microalgal species. The dinoflagellate *A. catenella* (in 2006) had the lowest genotypic richness with 1964 distinct genotypes, while the other dinoflagellate *A. ostenfeldii* was probably represented by more than 2 million distinct genotypes in the Åland archipelago (Table I). Both *A. catenella* and *P. multistriata* experienced shifts in genotypic richness between years according to our simulations.

**Fig. 5:**
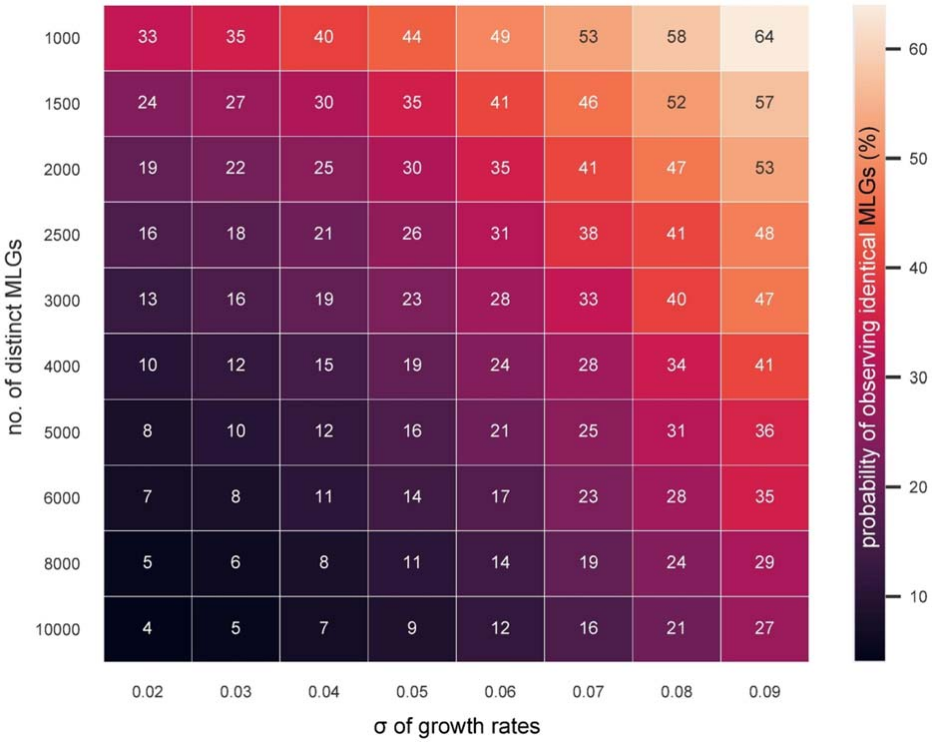
Heatmap visualizing the probability (%) of observing identical MLGs of *Ditylum brightwellii*, indicated by the color gradient, depending on the initial number of distinct MLGs and the intraspecific variability in growth rates (σ). μ = 0.18, sample size = 590 isolates, exponential growth period = 20 days, initial cell number per genotype = 1–100.

As the simulations with generic species (Fig. 3) indicated a large impact of intraspecific variability in growth rates on the probability of observing identical MLGs, we also ran simulations on all combinations of a range of sample sizes and standard deviations of the normally distributed growth rate function in *D. brightwellii*. Assuming the same sample size (590 isolates), the probability increased with the variation in growth rates among genotypes (σ of growth rates) (Fig. 5). With σ ≥ 0.07, the probability of observing identical MLGs usually exceeded 20%.

### Estimating genotypic richness with Chao1 and ACE indices

Based on the capture-recapture principle, the genotypic richness estimates from Chao1 and ACE indices were usually much lower than the estimates from our simulations (Table I). Similar to our model output, *A. catenella* and some *P. multistriata* populations from 2013 had the lowest estimated genotype richness while *S. marinoi, A. ostenfeldii* and *P. multistriata* in 2010 had the highest. Chao1 and ACE indices approach the results of our simulation in *D. brightwellii* likely due to the very high sample size in that study.

**Fig. 6:**
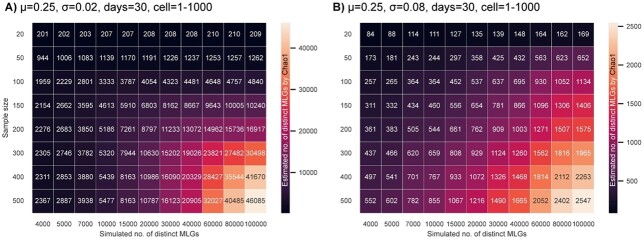
Heatmap visualizing mean Chao1 indices of simulated populations, indicated by the color gradient, with known genotypic richness depending on sample size. The two generic populations (A & B) are characterized by different amounts of intraspecific variability in growth rate (σ), while the mean growth rates (μ), exponential growth periods (days) and the initial cell concentrations per genotype (cell) are the same.

These results were further supported by mean Chao1 indices calculated for simulated generic microalgal populations with known numbers of distinct MLGs. With high intraspecific variability in growth rates (σ = 0.08), Chao1 indices noticeably underestimated the number of distinct MLGs (Fig. 6A), while the extrapolation was more accurate in a scenario with lower variability (σ = 0.02) and large sample sizes (>100 isolates). Nevertheless, even in such a best-case scenario, genotypic richness estimates based on Chao1 indices accounted only for approximately 30% of the present distinct MLGs (Fig. 6B).

### The effect of marker set size on observed number of distinct genotypes

**Fig. 7:**
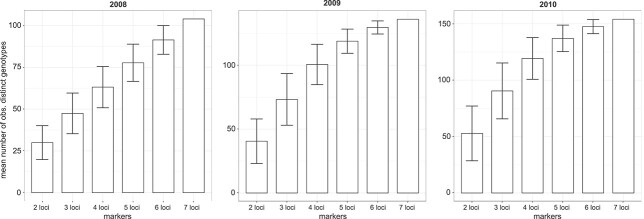
Mean number of observed distinct genotypes of *Pseudo-nitzschia multistriata* (Tesson *et al.*, 2014) depending on the number of applied genetic markers. Error bars indicate standard deviation from the mean calculated from all possible combinations of the number of selected microsatellites.

To gain insights into the effect of the number of loci used on the detectable amount of distinct genotypes, a rarefaction analysis was applied to the yearly microsatellite data from Tesson *et al.* (2014). In both 2009 and 2010, the mean number of observed distinct genotypes slowly leveled off around 130 and 150 genotypes respectively, with an increasing number of microsatellites (Fig. 7). In contrast, in 2008, the mean number of detected distinct genotypes increased with each additional locus (Fig. 7).

## DISCUSSION

The transient growth model constructed in Python 3.7.6 shed light on factors contributing to the large variability in genotypic richness among different microalgal species. The probability of observing identical MLGs in field studies of microalgal populations was strongly impacted by intraspecific variability in growth rates and sample size. This study highlighted the relatively low probability of observing identical MLGs with sample sizes between 20 and 50 isolates, which is supported by many observations from population genetic studies of microalgae (Casteleyn *et al.*, 2009; Demura *et al.*, 2014; Paredes *et al.*, 2019; Sassenhagen *et al.*, 2018; Sassenhagen, Sefbom, *et al.*, 2015; Sefbom *et al.*, 2018; Sjöqvist *et al.*, 2015). However, in a scenario with low mean growth rates and very high intraspecific variability, likely leading to the extinction of some and dominance of other clonal lineages, high proportions of identical MLGs can be observed also with low sample sizes. Nevertheless, detecting differences in genotypic diversity in studies involving high numbers of samples across spatial or temporal scales might be challenging, as the isolate numbers must be balanced against the number of samples required for achieving high-resolution data sets. The low number of identified identical MLGs in many large-scale studies characterized by a low number of isolates (<100) per sample results therefore from undersampling of the genotypic diversity.

Although we attempted to use realistic model parameters for the algal species selected in this study by only including monitoring data, it still remains challenging to properly assess in-situ growth rates of different genotypes in microalgal populations. Growth rates of individual algal strains estimated from laboratory experiments are likely not representative of natural environments due to a lack of grazers and optimized growth conditions. However, individual growth rates likely vary more than the overall population growth rates suggest (Kremp *et al.*, 2012; Sassenhagen, Wilken, *et al.*, 2015), as such monitoring data represent composite growth rates of all individual cells in the population. The simulations showed that the probability of observing identical MLGs increased with an increasing standard deviation of the mean growth rates. The genotypic richness estimates in this study are therefore likely quite conservative, as a certain observed proportion of identical MLGs would correspond to an even higher number of distinct genotypes if the standard deviation among growth rates were larger (Fig. 4).

The genotypic richness estimated through simulations in this study varied between 2000 and 2 million distinct genotypes among the different microalgal species, exceeding estimates from previous studies (Rynearson and Armbrust, 2005). In case the simulated genotypic richness translates into similar amounts of phenotypic variability in these microalgal populations, this high plasticity might underpin high evolutionary fitness and facilitate future adaptations to changing environmental conditions. Although the genotypic richness estimates are much higher than common sample sizes for population genetic studies of microalgae, current approaches might still be able to accurately detect patterns of genetic diversity and population structure, as it is not necessary to detect all present alleles. Instead, population genetic analyses depend on detecting representative allele frequencies, which can be achieved without sampling rare alleles (Hale *et al.*, 2012).

The dinoflagellate *A. catenella* had the lowest genotype richness in both investigated years. Although the observed proportion of identical MLGs in this species was comparable to reports in the diatoms *D. brightwellii* and *P. multistriata* (summer bloom 2013), the low intraspecific variability in growth rates based on monitoring data from Salt Pond resulted in an even distribution of genotypes at the peak of the bloom and therefore a low estimated genotypic richness. Compared to other microalgal blooms, which can extend over several hundred square kilometers (Erdner *et al.*, 2011; Paredes *et al.*, 2019; Trainer *et al.*, 2020), the *A. catenella* population in Salt Pond is very restricted in space. The total number of cells and distinct genotypes in this population is thus likely lower than in blooms of many other microalgae. Unlike many other microalgae with high dispersal potential, the dinoflagellate population in Salt Pond also experiences very limited genetic exchange with other populations (Richlen *et al.*, 2012). Both factors differentiate the selected *A. catenella* population from the congeneric *A. ostenfeldii* population, which inhabits a much more open area in the Åland Islands archipelago in the Baltic Sea. The several thousand islands in the archipelago are likely to create numerous ecologically distinct niches in this part of the Baltic Sea, which might select for different local adaptations and an especially high genotypic richness in *A. ostenfeldii* in the region. High habitat variability including the existence of many micro-niches might thus enable the co-existence of many different genotypes, which can arise from frequent immigration of new genetic varieties or selection for a wide range of different physiological strategies. These factors might explain the surprising range of genotypic richness among species of the same microalgal genus.

The estimated number of distinct genotypes also varied substantially among the three selected diatom species. This wide range can in part be explained by the differences in estimated intraspecific variability in growth rates among the species. *D. brightwellii* displayed similar proportions of clones as *P. multistriata* in 2010 (18 vs. 13%), but its estimated genotypic richness was much lower due to the relatively lower amount of intraspecific variability in growth rates. In contrast, the extremely high genotypic richness in *S. marinoi* was mainly driven by the low proportion of observed clones (0.65%) in this species (Godhe and Härnström, 2010). Although the study of *P. multistriata* by Ruggiero *et al.* (2017) employed a higher number of microsatellite markers (22 vs. 8), they observed a much higher proportion of identical MLGs (8.2–81%), emphasizing the extreme genotypic richness in *S. marinoi* independent of the sensitivity of the chosen fingerprinting method. Comparisons of genotypic richness in *P. multistriata* in successive years highlighted large temporal variation in the number of distinct genotypes. This variability among years might be caused by different environmental conditions that impact the survival of certain clonal lineages during the winter. Furthermore, intraspecific variability in growth rates might differ among years and can distinctly shape the genotype composition at the peak of the bloom (see Fig. 3C) potentially causing the massive clonal expansion in autumn of 2013 (Ruggiero *et al.*, 2017).

Intraspecific variability in growth rates seems, thus, to play a major role in shaping genotype composition and the probability of observing identical MLGs in microalgal blooms. Besides factors such as population size, immigration rates and frequency of sexual reproduction, variability in growth rates among genotypes of the same microalgal species is also driven by environmental selection pressures that facilitate the growth of certain genotypes while suppressing others. A low proportion of identical MLGs observed in population genetic studies might therefore suggest the absence of strong selection pressures and therefore environmental conditions that are suitable for the growth of many different genotypes. In contrast, a high proportion of identical MLGs could indicate strong environmental selection pressure that is only beneficial for the growth of certain genotypes. Furthermore, some microalgal species might be more sensitive to changes in environmental conditions than other species resulting in potentially large differences in intraspecific variability in growth rates among species as observed in this study. In addition to environmental selection pressures, we also need to consider selection of specific genotypes by cultivation in laboratory conditions. Many isolates die during cultivation before genotyping is complete and are therefore not included in population genetic analyses. This process could bias our data in both directions by exclusion of dominant lineages that do not grow well in the laboratory on the one hand, and reduction of the overall genotypic diversity on the other hand.

As mentioned above, species-specific frequencies of sexual recombination also have a large impact on genotypic richness. Sexual reproduction events in microalgae are likely to occur in episodes and are context-dependent. Thus, the timing of population sampling is of importance. The timing may either over- or underestimate the mean genotypic richness in a population, as seen in the diatom *P. multistriata* (Ruggiero *et al.*, 2017). As sexual reproduction might occur less frequently in diatoms than in dinoflagellates, which often go through sexual recombination at the end of the growth season before forming resting cysts (Brosnahan *et al.*, 2014; D’Alelio *et al.*, 2009; Jerney *et al.*, 2019), the number of cells per genotype at the beginning of the growth season might vary among species. Furthermore, cyst banks established over many years could provide an additional advantage for well-adapted, native genotypes following the monopolization hypothesis (De Meester *et al.*, 2016, 2002), in which the first colonizers outcompete most allochthonous genotypes. Varying the number of initial cells per genotype in our simulations did not, however, have a strong effect on the probability of observing identical MLGs, neither in populations with very low, nor with very high intraspecific variability in growth rates. To notice a significant impact of priority effects, genotypes with higher initial cell numbers likely also require a higher growth rate to significantly impact the final proportions of genotypes in the populations. This particular combination could not be investigated with this model due to the random distribution of cell numbers and growth rates among genotypes in the simulations. 

The continuous discovery of new genotypes of *A. catenella, P. multistriata* (in 2010) and *D. brightwellii* throughout the sampling period (Fig. 2) suggests that not all of the clonal lineages present had been sampled. This apparent under-sampling of genotypic richness in most population genetic studies of microalgae likely limits the predictive power of capture-recapture approaches. Accordingly, the estimates of genotypic richness in the selected natural microalgal populations based on Chao1 and ACE indices were consistently lower than the estimations from our simulations. Calculations of Chao1 indices for simulated generic populations with known genotypic richness showed that this discrepancy largely results from the heterogeneous genotype abundance in microalgal populations due to high intraspecific variability in growth rates. In particular with small sample sizes, this heterogeneity causes a negative bias of the Chao1 estimator, which was derived as a lower bound, rather than as a point estimator (Chiu *et al.*, 2014). However, when studying microalgal populations with relatively low genotypic diversity, very high sample sizes, as attained by Rynearson and Armbrust (2005), seem to improve the predictive power of the Chao1 and ACE indices resulting in similar estimates as our simulations.

The presented estimates are based on observed proportions of identical MLGs defined by a chosen set of microsatellite loci. Most microsatellite studies utilize between five and 15 different loci (see references in Sassenhagen and Erdner, 2017). Individual strains that look identical based on e.g. seven of these loci might turn out to be distinct when additional loci are investigated, as indicated by the rarefaction analysis in this study (Fig. 7). This analysis also highlighted differences in sensitivity of the chosen genetic markers between years or populations. In 2009 and 2010, the seven microsatellite loci applied in Tesson *et al.* (2014) were likely suitable to properly describe the present genotype diversity, while a higher number of loci might have been desirable in 2008. The number of observed identical MLGs will therefore often depend on the depth of fgenetic analysis applied. With the rise of Restriction-site associated DNA sequencing (RADseq) and whole-genome-sequencing, higher amounts of genetic diversity will be revealed in microalgae. Such population genomic approaches will therefore change estimates of genotypic diversity in microalgal populations in the future. The presented simulation of genotype propagation throughout one growth season and the resulting observed proportions of identical MLGs depending on sample size and variation in growth rates represents a tool, with which we can visualize these changes in genotypic richness throughout the evolution of our molecular methods.

## CONCLUSIONS

The genotypic richness estimates realized in this study varied between 2000 and 2 million distinct genotypes among the selected microalgal species. Differences in bloom size, immigration rates, frequency of sexual reproduction and habitat variability might explain the wide range of genotypic richness among different species. Several factors also influence the intraspecific variability in growth rates, which appears to be a major driver of genotypic richness. Environmental conditions allowing the co-existence and growth of many different genotypes, diverse nutrient-uptake and metabolic strategies, and a wide range of different ecological niches likely contribute to equal growth rates of many different genotypes. Such equal growth rates result in an even distribution of the present genotypes after a period of exponential growth and a low probability of observing identical MLGs in microalgal blooms despite the dominance of asexual reproduction. The surprisingly high diversity in some microalgal species estimated with our model might underpin high evolutionary fitness and facilitate future adaptations to changing environmental conditions.

## Footnotes

1 The *Alexandrium tamarensis* species complex recently underwent taxonomic revision, which determined that *A. catenella* has nomenclatural priority over two synonymous names—*A. catenella* and *A. tamarense* (John *et al.*, 2014; Prud’homme van Reine, 2017).
